# Long-term effects of environmentally relevant doses of 2,2',4,4',5,5' hexachlorobiphenyl (PCB153) on neurobehavioural development, health and spontaneous behaviour in maternally exposed mice

**DOI:** 10.1186/1744-9081-7-3

**Published:** 2011-01-13

**Authors:** Marte Haave, Annette Bernhard, Finn K Jellestad, Einar Heegaard, Trond Brattelid, Anne-Katrine Lundebye

**Affiliations:** 1 National Institute of Nutrition and Seafood Research (NIFES), P.O. box 2029 Nordnes, N-5817, Bergen, Norway; 2 Department of Biology, University of Bergen, P.O. box 7803, N-5020, Bergen, Norway; 3 Department of Biological and Medical Psychology, University of Bergen, Jonas Lies vei 91, N-5009, Bergen, Norway; 4 The Norwegian Forest and Landscape Institute, Fanaflaten 4, N-5244 Fana, Norway

## Abstract

**Background:**

Polychlorinated biphenyls (PCBs) are widespread in the environment, human food and breast milk. Seafood is known to contain nutrients beneficial for the normal development and function of the brain, but also contaminants such as PCBs which are neurotoxic. Exposure to non-coplanar PCBs during brain development can disrupt spontaneous behaviour in mice and lead to hyperactive behaviour. Humans are chronically exposed to the highest relative levels of organochlorines in early childhood during brain development, though usually at doses which do not give clinical symptoms of toxicity. This study aimed to elucidate the developmental and behavioural effects of 2,2',4,4',5,5' hexachlorobiphenyl (PCB153) in mice, mimicking human exposure during gestation and lactation.

**Methods:**

Environmentally relevant doses of PCB153 were added to the experimental diets. Feed concentrations were approximately 0.5, 6.5, and 1500 μg PCB153/kg feed, representing a realistic and a worst case scenario of frequent consumption of contaminated fish. The study also investigated the effects of maternal nutrition, *i.e. *a standard rodent diet versus a high inclusion of salmon. Mice pups were examined for physical- and reflex development, sensorimotor function and spontaneous behaviour from five days after birth until weaning. A selection of pups were followed until 16 weeks of age and tested for open field behaviour and the acoustic startle response (ASR) with prepulse inhibition (PPI). Blood thyroid hormones and liver enzymes, blood lipids and PCB153 content in fat were examined at 16 weeks. Statistical analyses modelled the three way interactions of diet, PCB exposure and litter size on behaviour, using generalized linear models (GLM) and linear mixed effect models (LME). The litter was used as a random variable. Non-parametric tests were used for pair wise comparisons of biochemical analyses.

**Results:**

Litter size consistently influenced pup development and behaviour. Few lasting PCB153 related changes were observed, but results indicated effects on synchronization of physical development. Perinatal PCB153 exposure appeared to reduce habituation and cause aggression in males, though not statistically significant.

**Conclusions:**

Litter size and maternal diet influenced physical development and function more than PCB153 in perinatally exposed mouse pups and supports the developmental importance of maternal care and the social environment.

## Background

Polychlorinated biphenyls (PCBs) were produced in large quantities before their extensive ban in the 1970s and early '80s. Their persistence to degradation and their global dispersion by air and ocean currents have made them omni-present in the environment, including food and breast milk [1-4]. Human PCB exposure is typically in the form of long term dietary intake of relatively low doses. The highest exposure to organochlorines occurs during the first years of life in breast-fed infants [1,2,4-6] which coincides with the period of rapid brain growth and maturation, also called the postnatal transition period [7] or Brain Growth Spurt (BGS) [8]. This is the period which has been shown to be vulnerable to organohalogen exposure in animal models [9-14]. In utero exposure to PCBs has furthermore been linked to cognitive and behavioural impairment in humans [15], and high levels of organochlorines in breast milk have been related to reduced neurological optimality in neonates [16]. In animals, exposure of young mice to low doses of di-ortho PCB, caused increasing hyperactivity and altered spontaneous behaviour with age [12,13]. Mechanisms of effect of PCB exposure on cognitive development are not fully elucidated, and have been found to include structural as well as hormonal changes, such as reduced density of cholinergic and muscarinic receptors [11,12,17], and thyroid disruption in highly exposed animals and humans [18-20]. Several mechanisms of effect have been suggested for PCB related thyroid disruption leading to cognitive effects [21].

Animals exposed to di-ortho PCBs during BGS also show behavioural patterns which have been compared to both attention-deficit/hyperactivity disorder (AD/HD) and the progress of Alzheimer's disease [12]. The non-dioxin like (NDL) PCBs like PCB153 have traditionally not been perceived as very toxic, since they do not act through the same pathway as dioxins. Investigations show that the toxic effect of these PCBs is not linked to their dioxin-like properties (coplanarity) [22]. Non-coplanar PCBs and the similar PBDEs have been observed to be neurotoxic and cause membrane disruption or behavioural changes whereas coplanar and dioxin-like compounds do not cause an effect [10,23,24]. This implies that also NDL congeners should be considered in toxicological evaluations. The recognition of the potential neurotoxic effects of NDL PCB congeners has lead to advice from the European Food Safety Authority (EFSA) that the NDL PCBs should be lowered as much as possible, and that they should be included in risk-benefit evaluations and monitoring programs to increase consumer safety [25].

However, established test paradigms that can reliably extrapolate results from animals to humans, or among animal models are rare [26,27]. One well conserved response in mammals which has been deemed appropriate for assessment of neurological aberrations, and possibly extrapolation to humans, is the startle response with prepulse inhibition (PPI) [28]. Few experimental studies examine the detrimental effects of PCB concentrations or exposure modes relevant to humans. Seafood is a rich source of many beneficial nutrients important for normal brain development and thyroid function [29,30] as well as a major source of dietary contaminants for seafood consumers [31-33]. The general public, and in particular pregnant women are advised to increase their intake of seafood and fatty fish due to its nutritional value [34-36]. The importance of nutrition has also been considered in recent epidemiological studies where the cognitive functions have been examined in children in relation to PCBs, certain nutrients and breast feeding [37]. Nutrients have also been found to protect against the effects of several environmental contaminants by means of counteracting oxidative effects [38], sequestration [39], stimulation of metabolism [40] or by reducing the uptake of the contaminants [41,42]. Dietary selenium supplementation in the mother has also been shown to ameliorate detrimental effects of methylmercury in murine offspring [43]. This suggests that the nutritional composition of the diet during gestation and lactation may affect the experienced toxic exposure of both dam and offspring, and their ability to tolerate the exposure.

The potential developmental effects of PCBs relevant to human exposure need to be examined using relevant doses and exposure models. The aim of this study was to evaluate possible adverse developmental effects in young mice exposed to PCB153, in an exposure model relevant for humans, and to investigate the influence of maternal intake of potentially ameliorating seafood nutrients. This includes the gestational and lactational exposure of offspring via cord blood and breast milk during the vulnerable stages of BGS.

## Methods

The experiment and the animal facilities were approved by the National Animal Research Authority (FDU, Norway). The study conforms to the requirements of the Norwegian National Committee for Animal Welfare, which closely conforms to the European Convention for the protection of Vertebrate animals used for Experimental and other Scientific Purposes (Council of Europe no. 123, Strasbourg 1985).

### Nutrition

The experimental diets were produced in house according to the AIN-93 G Rodent diet to meet 1995 NRC Rat/Mouse Reproduction, Gestation and Lactation Values (DYETs Inc. formulation #110800). In order to investigate the effects of nutrition, two sets of diets were produced with similar contaminant exposure but different nutritional composition. Thus one diet was produced with and one without inclusion of Atlantic salmon (*Salmo salar; *Additional file 1). Briefly, the casein-based diet used casein sodium salt (Sigma Aldrich Inc.) as the main source of protein, and soy-bean oil as the sole source of lipids. The fish-based diet used 15% (per weight) freeze dried Atlantic salmon raised on vegetable feeds [44] which gave low levels of environmental contaminants [45]. The salmon was as a source of both protein and lipids to the fish diet, and the fish-based feeds were added casein and soy-bean oil to reach the desired concentrations of 17% protein and 10% fat. The same concentrations of protein and lipid were obtained for all diets, analysed by accredited methods at NIFES (Additional file 2).

### Spiking and doses of PCB153

To mimic human exposure animals were exposed to PCB153 via *ad libitum *food intake, through diets spiked with PCB153 (Chiron AS, Norway). Dietary PCB concentrations were verified by Gas Chromatography/Mass Spectrometry (GC/MS) in SIM mode, performed at NIFES by accredited methods based on previous publications [46,47]. One fish and one casein diet were left unspiked for control, while PCB153 was added in high or low concentrations to both fish and casein diets, producing a total of six diets: Casein Control, Casein Low PCB and Casein High PCB, Fish Control, Fish Low PCB and Fish High PCB. The spiked low dose diet aimed at a PCB153 concentration similar to the concentration typically found in farmed Atlantic salmon from Norway [48], where PCB153 is the most prevalent congener. The exposure to PCB153 from the diets with the spiked high concentrations represented a worst case scenario with repeated consumption of fish with extremely high levels of PCB153. The intake of food was monitored by weighing any uneaten food daily, and calculation of the dose per kg body weight (BW) was done for gestation and lactation. Calculated doses ingested by the dams in the high dose groups were similar to the doses previously shown to produce persistent changes in spontaneous behaviour after single exposure on Postnatal day (PND)10 [13]. Dams with 30 g body weight consuming a maximum of 12 g feed with 1500 ngCB153/g feed during lactation would have a peak daily intake of PCB153 of 1.7 μmol/kgBW*day. Feed and thus contaminant intake by the dams peaked around PND11, which coincides with the BGS in mice [8]. The estimated exposure of each pup would be highly influenced by pup body weight and number of pups per litter. The lactational transfer to pups is also a relevant route of exposure for humans, and includes transfer of metabolites of the parent compound from the dam. Exposure to potentially toxic metabolites would not be obtained by direct exposure of the pups, as pups have limited metabolic capacity [49].

### Animal model and housing

Mice are much used as a model for developmental neurotoxicity [10-14,17,50-62].

**Figure 1:**
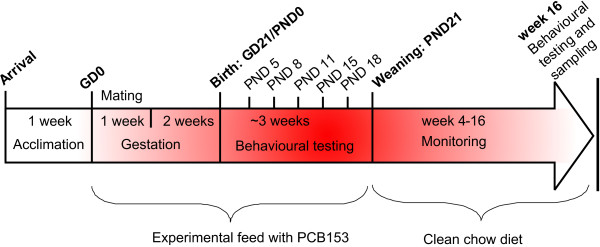
**Experimental outline**. The experiment was performed during 16 weeks. Mice offspring were treated and handled as indicated. GD = Gestation day, PND = Postnatal day.

54 female BALB/c mice from Charles River Inc. (Germany) were housed in groups of three in a large rat cage (Eurostandard Type IV) evenly distributed within racks to compensate for any environmental variation. Housing conditions were standardized to 25 ± 2°C, 55 ± 5% relative humidity, and 12:12 hr light-cycle, lights on during the day. To promote natural behaviour and alleviate stress in the animals, cages were equipped with the following environmental enrichment: a transparent polycarbonate mouse igloo with an activity wheel (Bio-Serv), a "mouse loft" (Tecniplast), aspen chewing sticks and dust-free "Sizzle-Nest" (Scanbur). Dams were acclimated to control diets *ad libitum for *one week before mating, and experimental feeds were given *ad libitum *during mating, gestation and lactation (until PND19; Figure 1). The first day of mating was denoted gestation day (GD)0. Mating was performed within one week, with one male per three females. The males were rotated after the first oestrus cycle in case of infertility in the males, which has been observed previously (data not shown). On GD16 females were separated into single cages (Eurostandard Type III H) in order to monitor each litter separately. Cages were checked twice daily for litters from GD18. The day of birth was denoted PND0. Pups were weighed and handled every third day from PND5 until PND18 (Figure 1, Table 1).

**Table 1:** Observations of physical development and behavioural tests in murine offspring

Physical markers of development	PND5	PND8	PND11	PND15	PND18	Week 16
Weight	**+**	**+**	**+**	**+**	**+**	**+**
Freeing of pinnae	**+**	**+**				
Fur development	**+**	**+**				
Incisor eruption		**+**	**+**	**+**		
Eye opening			**+**	**+**		

**Behavioural testing**	**PND5**	**PND8**	**PND11**	**PND15**	**PND18**	**Week 16**

Grasping reflex	**+**	**+**				
Righting reflex	**+**	**+**				
Climb/hang test	**+**	**+**	**+**			
Auditory startle		**+**	**+**	**+**		
Visual placing				**+**		
Cliff drop aversion				**+**		
Forelimb strength				**+**	**+**	
Open field					**+**	**+**
Startle reflex (ASR/PPI)						**+**

Behavioural tests were performed on all pups in a litter from postnatal day (PND) 5 to PND18 and at 16 weeks of age. +: Signifies that the test was performed that day.

Most animals were sampled on PND19. For the high level PCB groups and the Fish Control group, one male and one female sibling per litter were weaned on PND21, and monitored until week 16. All pups from low dose groups were sacrificed on PND19 due to a low number of litters in the Casein Low PCB group. All animals were marked for identification and tracking of developmental history. Pups were given water and standard rodent diet in pellets (chow, 10% fat) *ad libitum *after weaning. Providing clean chow during development would imply that any observable toxic effects must have been caused by the exposure during early development. Pups were weighed weekly and inspected daily from Monday to Friday. Aggressive males were separated into individual cages. All males were separated one week before behavioural testing in week 16, to minimize the differences in caging conditions and stress level before the final behavioural test.

### Behavioural testing

From PND5 to PND18 the pups were observed and tested for physical markers of development, reflexes and motor-function (Table 1). Behavioural tests were done at the same time of day for each age-group, in a dedicated room with the same temperature and lighting as the housing conditions. All pups in a litter were tested by a single experimenter who was blinded to the treatment. Where possible, at least one pup was left in the cage at any time during testing to relieve maternal stress. On PND5, -8 and -11 all pups were tested for behavioural reflexes, based on the Fox battery [7] with modifications previously described [63]. The individual tests were chosen based on experience from previous trials [43,64]. The following tests were performed as described by Folven *et al. *[43]: righting, hind limb grasp, a combined hang/climb-test and auditory startle reflex, with a small modification for the startle reflex test which was tested using a metal "click-box" that produces a sharp sound without visible movement. Except for hang/climb all reflex tests were scored 0-1, where "1" is the display of characteristic behaviour [43]. For the hang/climb-test, graded levels of hanging and climbing were scored. Climbing was defined as moving and successfully replacing at least one fore- and one hind limb. The recordings were scored such that: 0 = no hang, 1 = 1-14 sec, 2 = 15-29 sec, 3 = 30-44 sec, 4 = 45-59 sec, 5 = > 60 sec, 6 = successfully climbing, replacing two limbs, 7 = successfully replacing three limbs, and 8 = successfully replacing all four limbs while climbing.

Additionally, pups were tested for visual placing and cliff aversion [7] after eye opening on PND15. Forelimb strength was measured for pups on PND15 and -18 by a grip strength meter (San Diego Instruments Inc., San Diego, USA), and on PND18 pups were tested for anxiety and spontaneous behaviour in an open field. The open field arena was a (42 × 42) cm black polyethylene arena with 25 cm high walls, enclosed by white sheets hanging from the ceiling, and lit by indirect lighting to create a uniform environment and exclude visual cues. For the data analysis the open-field arena was divided into two zones, a periphery, defined as a 7 cm wide area along the walls, and a centre zone defined as the (28 × 28) cm square in the centre. On PND18 the animal was placed in the centre of the field and latency to leave the centre was measured. In week 16 the animals were placed in an adjacent chamber, allowed to acclimate for two minutes, before a door to the open field was opened and latency to enter the open field was measured. On PND18 movement was recorded for three minutes and in week 16 for 10 minutes, using a digital camera and the behavioural tracking software SMART (San Diego Instruments Inc., USA). The tracking recorded the latency to leave the start zone (PND18 only), latency to enter the field (week16 only), total distance travelled, time spent resting, the number of entries into the centre, and the total time spent in centre and periphery, respectively. Also recorded was the distance travelled along the perimeter (% of total distance), and speed of movement in the perimeter. The arena was wiped with 70% ethanol and water between trials. The test order was randomized with respect to treatment group, litter and individuals in the litter. The test was performed at a consistent time of day.

### Complex behaviours: prepulse inhibition

The startle response is an unconditioned response to an audible, tactile or sensory stimulus. The response is plastic, in that it can be modulated depending on other external cues and repeated stimuli. Prepulse inhibition (PPI) is a phenomenon where a weaker stimulus prior to the main stimulus attenuates the response to a subsequent startle response. This modulation of the startle response is highly conserved in mammals, and has been used and discussed as a cross-species measure of sensorimotor gating [28,65]. Abnormalities in PPI have been linked to neuropsychiatric disorders and diseases like schizophrenia, Alzheimer's and Huntington's disease [28,65]. At 16 weeks of age, the animals were tested for acoustic startle response (ASR) with prepulse inhibition (PPI) using the "Startle box" (Med Associates Inc., USA). The mice were placed in a grid floor animal holder in the sound-attenuating cubicle, acclimated for five minutes with 60 decibel (dB) ambient white noise and thereafter exposed to 40 acoustic stimuli (Additional file 3). All stimuli were presented as 120 dB white noise signals over a 60 dB ambient white noise. Prepulses (73, 75, 80 or 85 dB) were presented in pseudorandom order 100 ms prior to the 120 dB main stimulus (Additional file 3). Inter-trial intervals varied randomly from 10 to 20 seconds. The ASR was defined as the peak amplitude after the 120 dB main stimulus, subtracted the peak amplitude caused by spontaneous activity for 200 ms prior to prepulse presentation. Mean PPI for each prepulse intensity was calculated as percent reduction in mean startle response with prepulse (ASR-pp) compared to the startle response without prepulse (ASR-simple, Additional file 3) according to the formula (ASR-pp/ASR-simple)*100-100. Habituation was calculated from the differences in mean response of the five last ASR-simple compared to the first five ASR-simple, according to the formula [(ASR block3-ASR block1)/ASR block1]*100-100. With these approaches, normal inhibition of the startle response and habituation produce high negative values, whereas lack of inhibition and habituation gives values close to zero, or positive values. PPI in untreated male BALB/c with this range of prepulse intensities has previously been shown to be from 40-60% [66]. Based on suspected gender differences in the PPI response a decision was made *a priori *to separate genders.

Shortly before the final sampling two males from Casein High PCB and one male from Casein Control were scanned with Magnetic Resonance Imaging (MRI) as a preliminary check for macro-anatomical changes in the brain. No apparent changes in ventricle size or gross brain morphology following the repeated perinatal PCB-exposure were seen, and the remaining animals were not scanned.

### Sampling

At PND19 most pups were euthanized and the liver was sampled and frozen at -20°C until PCB analysis. At 16 weeks of age, remaining animals were euthanized by exsanguinations. Blood was drawn from the heart with a syringe and mixed with 10 μl heparin (2.02 units/μl), immediately centrifuged at 4°C, 2500 rpm for 5 minutes and the serum frozen at -80°C until biochemical analyses (within two months). Liver enzyme analyses were performed on serum samples to assess liver damage. Blood sugar levels and blood lipids were also analysed in order to examine differences related to early toxic exposure with different maternal diets. Analyses were performed using a MAXMAT PL multipurpose diagnostic analyzer system (MAXMAT S.A. Montpellier, France). The following kits from DIALAB GmbH: Alanine transferase/Glutamate Pyruvate Transaminase (ALAT) with Pyridoxal 5' phosphate, Alkaline Phosphatase (ALP), total Cholesterol (Chol), Gamma-glutamyl transferase (GGT) and High Density Lipoprotein cholesterol (HDL); and from MAXMAT: Glucose (Glc), Lactate Dehydrogenase (LAD), and Triglycerides (TG) were used for serum analyses. Analyses of ALAT and GGT failed in a number of samples and were omitted from results. Two additional samples were omitted from the analysis due to outlying results caused by technical problems during sampling.

Additionally, serum was analysed for free Triiodothyronine (fT3), free Thyrosine (fT4) by RIA and Thyroid Stimulating Hormone (TSH) by IRMA, according to the manufacturer's specifications (DRG international, Inc). Only one replicate was analysed per sample due to limited amounts of serum.

Concentrations of PCB153 in pup livers on PND19 and abdominal fat in week 16 was analysed by Gas Chromatography/Mass Spectrometry (GC/MS) in SIM mode, based on the same methods as used to analyse the feeds [46,47].

### Statistical analyses

The statistical methods employed here keep the litter as the experimental and statistical unit. This is highly recommended when working with multiparous species, as pups from the same litter are more similar than pups from different litters [67,68].

### Statistical models

All analyses aimed to assess how the response characteristics were influenced by the independent factors "diet" (fish or casein), "PCB153 level" (control, low, high), and "litter size" (1-10 pups) and their interactions. Inclusion of the dam as a random factor kept the litter as the statistical unit, as recommended [67,68]]A Linear Mixed Effect Model (LME) [69]was employed to model influences on body weight, weight gain week 4-16 (corrected for autocorrelation), Hepatosomatic Index (HSI = Liver weight*100/body weight), forelimb grip strength, total distance travelled in the field, latency to leave the start zone (PND18) and latency to enter the field (week 16 only). The LME procedure accounts for the pups being grouped by the dam. For the reflex responses (PND5-18) a generalized linear model (GLM) with quasi-/binomial distribution was employed. The GLM model applied the response from each pup as a trial for the dam. In this way information about the number of pups tested (trials) and the number of successful scores in the litter was included. Inclusion of the litter size as an independent variable provided a statistical test for the significance of the litter effect [68]. Additionally, in this model the dam was included as a random factor representing the litter, due to differences in maternal care. For the Open Field and the hang/climb test, scores were measured as percent of maximum and compared using a variant of this model (GLMM), similar to the LME procedure, but allowing for binomially distributed responses [70]. All models adjusted for uneven variance of the residuals (heteroscedascity). The model selections were obtained from backward elimination. Due to the reproductive failure in the Casein Control group, a full analysis including all possible terms was not possible. The biological signal was then assessed through two separate models instead; model I used the influence from and interaction between litter size, diet and two levels PCB (low or high) only, disregarding the control groups due to the missing Casein Control. Model II investigated the two-way interaction of litter size and PCB153 level among the fish groups (Fish Control, Fish Low PCB and Fish High PCB). Where comparison of single groups was warranted, Tukey HSD Post hoc test was used. Pearson's product-moment correlation test was used to test the correlation between body weight and litter size in pups.

The above statistical analyses were performed by the statistical software R [71]. SPSS 15.0 for windows (SPSS Inc., Chicago, IL, USA) was used for the nonparametric comparisons of reproductive success, biochemical analyses and for analysis of PPI. The non-parametric Kruskal-Wallis test of independent samples, followed by Mann-Whitney U pair wise comparison were used for comparison of means. Due to small sample sizes the "Exact significance-test" was used. Significance levels were set at alpha = 0.05 for all analyses.

Because of missing data, two litters were excluded from the analysis of eye-opening: One litter in Fish High PCB with 7 pups on PND12 and one litter in Casein High PCB with 7 pups on PND13. Two litters in Fish Low PCB with only one pup were excluded from comparisons of feed intake. Females were excluded from the Open Field analyses in week16 due to technical difficulties performing the test. Males that died before week 16 were excluded from comparisons of body weight from week 4-16.

## Results

### Comparability among groups and litters

**Figure 2:**
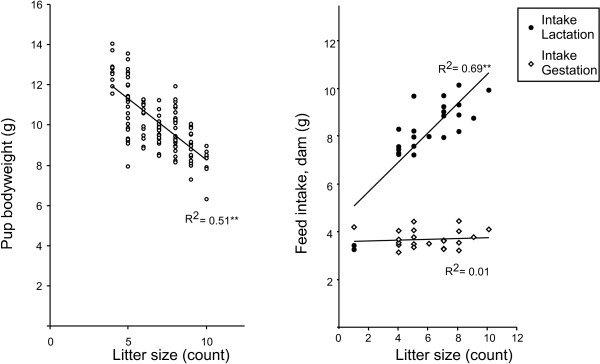
**influence of litter size on pup body weight and maternal feed intake**. Left panel: Negative correlation was observed between pup bodyweight and litter size. Right panel: Positive correlation was observed between maternal feed intake during lactation and litter size. Feed intake during gestation was not affected by litter size. **: p < 0.001.

There were no consistent, exposure related group differences in body weight or mean feed intake among dams (Table 2). Reproductive success and litter size was not different among groups that produced viable litters. The Casein Control group did not produce any viable litters. The cause for the reproductive failure is not know, but may reflect possible external stressors the first few days after birth, which by chance affected this group the most. Several viable litters were born in other treatment groups on the same day. All diets were found to contain 10% fat and 17% protein. The vitamin supplements used were identical for all feeds. Analysed concentrations of PCB153 in feeds are given in Table 2. Feed intake by dams increased from approximately 3.5 g/day during early gestation to a maximum of approximately 12 g/day during lactation. The feed intake by dams during lactation was strongly correlated with litter size (p < 0.001; Figure 2). The feed intake during lactation and hence the calculated intake of PCB was significantly higher for Fish High PCB than for Casein High PCB (Table 2).

**Table 2:** Feed concentration, body weight, reproductive output and feed intake by reproducing dams fed PCB153 in a fish or a casein-based diet.

Diet (n)	PCB153 ng/g feed	Initial BW dams	Final BW dams	Repr. succ %	Litter size	Intake gestation (g/day)	Intake lactation (g/day)	Gestation dose (μg/kgBW*day)	Lactation dose (μg/kgBW*day)
Casein Control (0)	0.25 ± 0.12	n.d	n.d	0	n.d	n.d	n.d	n.d	n.d
Fish Control (3)	0.50 ± 0.07	28.9 ± 0.5	30.4 ± 0.2	33	4.7(4-5)	3.6 ± 0.2	7.7 ± 0.4	0.05 ± 0.00	0.12 ± 0.00
Casein Low PCB (3)	6.53 ± 0.12	29.1 ± 0.5	31.1 ± 0.9	33	6.3 (5-9)	3.6 ± 0.1	8.3 ± 0.7	0.73 ± 0.06	1.72 ± 0.23
Fish Low PCB (5)	6.57 ± 0.67	29.6 ± 0.8 (7)	31.3 ± 0.6 (7)	78	7.0 (4-9)	3.6 ± 0.1	8.6 ± 0.5	0.69 ± 0.03	1.63 ± 0.11
Casein High PCB (6)	1400 ± 60	28.4 ± 0.9	31.3 ± 1.5	56	5.8 (4-8)	3.9 ± 0.2	8.1 ± 0.3*	170 ± 10	350 ± 20*
Fish High PCB (5)	1500 ± 300	30.0 ± 1.0	29.6 ± 0.7	56	6.8 (4-10)	3.5 ± 0.2	9.2 ± 0.3*	160 ± 10	420 ± 20*

Data are presented as mean ± SEM. Number of litters per group in brackets. Four litters were born in the Casein Control group, but no pups lived until postnatal day 5. Initial BW = BW after acclimation, final BW = BW end of weaning. Repr. succ: = percent successful reproduction in relation to the number of mated females. Litter size is given as mean (min-max). Feed intake during gestation (GD0-21) and lactation (PND0-21) was based on daily monitoring of feed intake. Daily doses were calculated based on average feed intake in grams and PCB153 concentrations in feeds, rounded off to the nearest ten ng/g for the high levels. Two females with single pups in Fish Low PCB were excluded from the calculation of litter size, feed intake and dose during gestation and lactation, but were included for comparisons of initial and final body weight (BW) of reproducing dams. n.d: No data due to reproductive failure. *: Groups that share a symbol are significantly different (Student's *t*-test: p < 0.05).

### Analyses of PCB153

Concentrations of PCB153 in pup livers at PND19 reflected maternal dietary intake. Pup liver PCB153 concentrations (Mean ± SEM) were 2.1 ± 0.5 ng/g, 9.6 ± 0.4 ng/g, 15.6 ± 5.7 ng/g, 2200 ± 300 ng/g and 1800 ± 200 ng/g ww for Fish Control, Casein Low PCB, Fish Low PCB, Casein High PCB and Fish High PCB respectively.

### Growth and development

**Figure 3:**
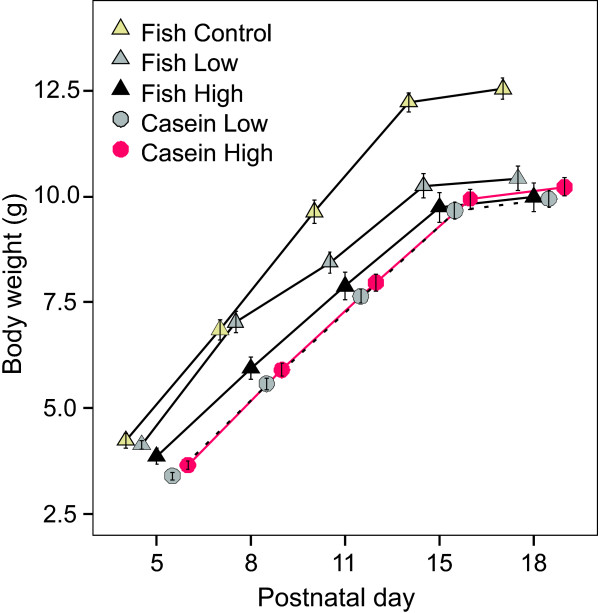
**Body weight gain in offspring during lactation**. Body weight was measured on Postnatal day 5, -8, -11, -15 and -18. Each point with error bars represents the mean body weight ± SEM per group, in pups of both genders.

**Figure 4:**
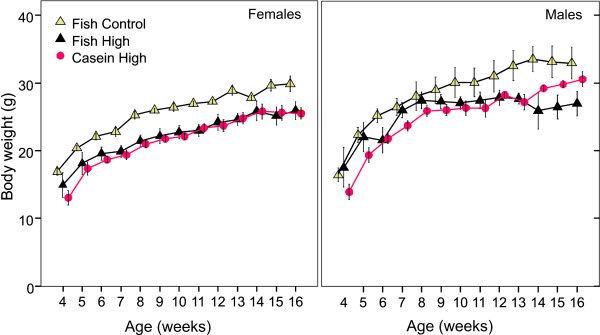
**Body weight gain in juvenile mice week 4 to 16**. Each point with error bars represents the mean body weight ± SEM in female (left panel) and male (right panel) BALB/c mice exposed to PCB153 during gestation and lactation. The data include animals that survived until week16.

Pup body weight was monitored from PND5-18 (Figure 3). Body weight of males and females are reported separately from week4 to week16 (Figure 4). The Fish Control group generally had a higher body weight than pups in the PCB exposed groups. This trend persisted from lactation until week16, but was significant only in week 16. The mean litter size of the Fish Control group was slightly less than the Casein High PCB and Fish High PCB groups (Table 2). The pup weight gain during lactation was mainly influenced by litter size (Figure 2) and was not significantly affected by the protein source (fish or casein) or the PCB level.

Females in the Fish Control group were significantly heavier than females in Casein High PCB (p < 0.01) at week16 after correction for litter size (Figure 4, left panel). LME (adjusted for repeated measurements) demonstrated that the initial litter size had an effect on male and female body weight until adulthood (p < 0.05 and p < 0.001) respectively.

### Developmental effects of litter size, maternal diet and PCB exposure

In the present study, the litter size was a prominent and recurring factor which affected the behaviour and development in a predictable and consistent manner. Physical development, such as body weight, fur development on PND5, incisor eruption on PND11 and eye opening on PND14 were negatively related to litter size, with consistent and significant effects or trends in the two different factorial models (data not shown). Regarding reflex development, a higher litter size significantly reduced the success rate in the hind limb grasp test (PND5), the hang/climb test (all days) and the startle reflex (PND11) in both factorial models (p < 0.05) Results for the forelimb grip strength were ambiguous. Statistical analyses by model I showed that a maternal fish diet was related to higher body weights in pups on all days from PND5 to PND18 (p < 0.05) after correction for the effect of litter size. The higher body weight may be the reason why a maternal fish diet was also related to more fur on PND5 (p < 0.05), and tended to give more pups with erupted incisors on PND11 (p < 0.1) as well as more pups with a startle response on PND15 (p < 0.1).

PCB exposure was not significantly related to body weight, growth or behavioural development. However, the timing of incisor eruption and eye opening appeared less synchronized in the PCB exposed pups than the controls. Comparing to Fish Control, the PCB153 exposed groups had earlier incisor eruption than the control group, but by PND11 the Fish Control was the only group with full development of the trait. Similarly, pups from Fish Low PCB and Fish High PCB had started eye-opening on PND12, whereas the pups from the Fish Control group had not. By PND14 there was no difference among groups.

### Spontaneous behaviour in the open field PND18

The open field used for assessment of spontaneous behaviour, anxiety and reactivity at PND18, showed differences in spontaneous behaviour among genders. At PND18 males spent more time in the centre of the open field, spent less time moving fast and travelled a shorter distance than females (all groups compared, p < 0.001, p < 0.05 and p < 0.05 respectively). No other gender effects were seen. For the remaining analyses genders were therefore grouped.

On PND18 the fish groups left the central starting zone significantly faster, and spent more time moving fast than casein groups (p ≤ 0.01 and p < 0.05, respectively). A trend towards lower resting time in the fish groups than the casein groups was also seen (p < 0.08), indicating higher reactivity in the fish-groups regardless of PCB exposure. No differences were observed for total distance travelled; time spent moving slow and fast, time spent resting or permanence time in the centre or the periphery.

### Open field in week 16

Similar to PND18, in week 16 males from the fish groups entered the open field significantly faster than the casein group (p < 0.01). No effects of PCB exposure were seen on the latency to enter the field or other parameters tested.

### Acoustic startle response with prepulse inhibition in week16

PPI tests showed large variability within the groups in both genders. The small sample size and large variance precludes statistical analyses, and the PPI test would need to be repeated with larger groups for conclusive results, and to determine possible diet, PCB exposure or litter effects. Although there were no significant differences among groups or genders, certain aspects of the findings deserve mentioning. This study shows that among the males, both of the PCB exposed groups showed consistently less prepulse inhibition than the controls (Additional file 4), although not significantly different. The reduction in startle response in exposed males was less than previously seen for BALB/c [66]. However exposed groups were not significantly different from the control group, and conclusions cannot be drawn.

### Habituation to the acoustic startle response

**Figure 5:**
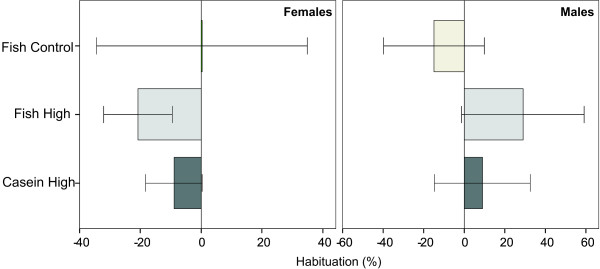
**Habituation to the acoustic startle stimuli in week 16**. Reduction in startle response at the end compared to at the start of the trial. The bars and the error bars represent the percent habituation (mean ± SEM). A negative value indicates a normal habituation response, with a lower startle response at the end than at the start of the trial. Sample sizes were (females/males): Fish Control: n = 3/3; Fish High: n = 4/5; Casein high, n = 6/4.

A reduced startle response in the last set of simple stimuli compared to the first set of simple stimuli indicates habituation, which is the expected response to repeated stimuli. Noticeably, in males, both of the PCB exposed groups showed the opposite response to habituation (Figure 5). The mean startle response was in fact higher in the last block of stimuli than at the start, indicating increased fear in that group, and no adaptation to the situation. The large individual variability and the small dataset render conclusions difficult.

### General observations of behaviour in PCB exposed males

In week 4-16 all female groups and the male controls had 100% survival. Mortality was only observed in PCB exposed males. By week 12 the males in the Casein High PCB group had a survival of 67% (4/6) and by week 13 males in the Fish High PCB group had a survival of 83% (4/5; Additional file 5). No deaths were caused by male aggression. However, all males in the Casein High PCB group were separated into single cages since they developed aggressive behaviour.

### Biochemical analyses of PCB concentrations, blood lipids and liver enzymes week 16

PCB153 was analysed in abdominal fat in week 16 to elucidate if maternal diet during gestation and lactation had an effect on pup metabolism and lasting body burdens of PCB153. After 12 weeks on a standard chow diet, groups still had PCB153 levels that reflected the maternal PCB intake during gestation and lactation. PCB153 concentrations were, Fish Control: 11.4 ± 2.7 ng/g ww, Casein High PCB: 7000 ± 600 ng/g ww and Fish High PCB: 7000 ± 700 ng/g ww (Mean ± SEM, high level groups rounded off to the nearest 100 ng/g ww). Serum contents of blood lipids, liver enzymes and glucose showed minor differences only in levels of TG among females (Additional file 6). There were no differences in blood lipids, liver enzymes or glucose among the males. For the females in week 16, the Fish Control group had the highest HSI (Table 3), which coincided with a higher body weight of females in the Fish Control group (Figure 3).

**Table 3:** Group size, body weight and hepatosomatic index for male and female offspring

Gender	Group	N	BW PND21	BW Week16	HSI Week16
female	Fish Control	3	16.9 ± 0.5	29.9 ± 1.1	4.97 ± 0.14^a^
	Casein High PCB	6	13.0 ± 1.1	25.5 ± 0.8	4.36 ± 0.10^b^
	Fish High PCB	5	14.9 ± 1.8	25.9 ± 1.3	4.65 ± 0.20^ab^

male	Fish Control	3	16.6 ± 1.0	33.0 ± 2.3	4.38 ± 0.20
	Casein High PCB	4	14.0 ± 1.2	30.6 ± 1.1	4.87 ± 0.38
	Fish High PCB	4	17.6 ± 3.1	27.0 ± 1.8	4.65 ± 0.62

The animals were exposed to PCB153 throughout gestation and lactation, via maternal consumption of spiked feeds based on fish or casein. High dose feeds (Fish High PCB and Casein High PCB) contained ~1500 μgPCB153/kg feed. Data are presented as mean ± SEM of offspring that survived to week16.

HSI = Hepatosomatic index, BW = body weight, PND = Postnatal day. Groups with different superscript letters are significantly different, within the same gender (Mann-Whitney U, p < 0.01).

### Thyroid status

**Figure 6:**
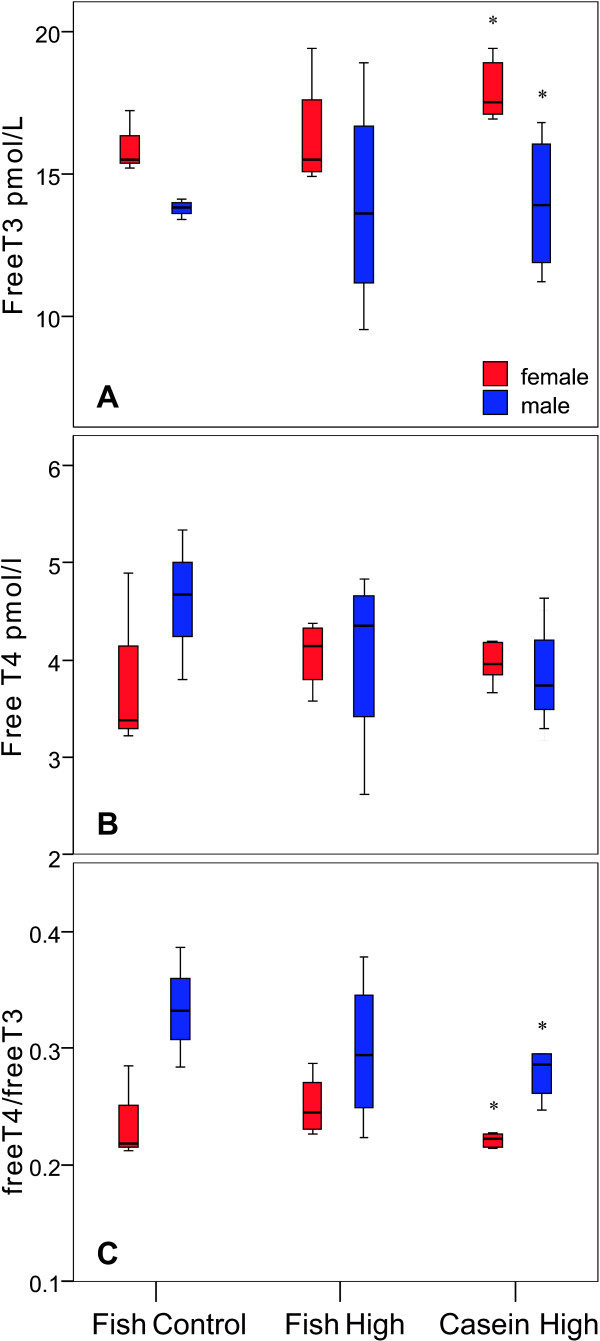
**Thyroid hormones in serum at week16 in murine offspring**. Hormone concentrations of free triiodothyronine (fT3, upper panel), free thyroxine (fT4, middle panel) and the ratio fT4/fT3 (lower panel) in pups exposed to PCB153 via gestation and lactation. The boxes show the median and the inter-quartile range. * indicates significant difference between genders (Mann-Whitney U pair wise comparison, p < 0.05).

No differences in fT3, fT4 or the ratio fT4/fT3 were found among groups (Figure 6). Comparing the genders, males generally had lower fT3 and higher ratio fT4/fT3 than females, which was statistically significant in the Casein High PCB group only (p < 0.05). TSH was below the level of quantification (0.2 pg/L) for all samples, so comparisons could not be made.

## Discussion

The study aimed to elucidate if a worst case scenario with high maternal PCB153 intake through fish consumption during brain development can be expected to give any observable behavioural or developmental effects. Maternal nutrition is important for offspring development, and fish is also a known source of many beneficial nutrients. The possible ameliorating effect of a maternal high fish diet was hence compared to a similar intake of PCB153 in a diet without fish.

Early pup development and behaviour as well as adult behaviour and health were assessed as toxic effects have previously been shown to manifest with increasing age[10-12].

The observations of this study suggested that continuous maternal exposure to the single PCB153 congener during gestation and lactation had little effect on parameters of physical and neurobehavioural development in the offspring, but that survival and behaviour in early adulthood might have been compromised. The study suggested that PCB153 exposure might have affected the synchronization of physical development, such as eye opening and incisor eruption. This tendency to disturbed synchronization of development has previously been observed in rats [72]. Disturbances in teeth development has also been observed in children exposed to PCBs during the so-called Yusho disasters where PCB contaminated rice-bran oil was ingested [73,74]. Nutritional effects on pup development were also present as trends; as pups of dams fed the fish diets had an accelerated development of physical traits, increased auditory startle reflex, and were more active in the open field compared to casein fed pups. Neither diet nor PCB exposure greatly affected development until adulthood, but the maternal fish diet may have caused increased body weight in pups. PCB exposure may have increased the mortality of the juvenile offspring in males. However, the biochemical and behavioural evidence of lasting adverse effects of perinatal PCB153 exposure in adult mice were scant, possibly as a cause of the low sample size. The biochemical and behavioural data from week 16 are thus inconclusive.

### Nutritional aspects

Considering the nutritional aspect of the study, all diets had the same levels of nutrients and fulfilled requirements for vitamins, minerals, protein and lipids (Additional file 7). An effect of nutrition on physical development has previously been seen with selenium supplementation, which increased fur development in neonate mouse pups [43]. It is possible that the more nutritionally complex fish diets, which contained both fish, casein, fish oil and soy-oil, provided a more optimal nutrient composition which was beneficial during early development. However, without a demonstrable adverse effect of PCB153 any possible ameliorating effects of fish could not be ascertained.

### Neurobehavioural tests

Previous studies have documented the validity and efficiency of the neurobehavioural tests used, and have detected neurobehavioural differences in mouse pups using a similar test battery [43,64]. The fact that subtle changes related to litter size were consistently detected in a predictable manner underlines the sensitivity and utility of the statistical methods. The effect of litter size, when present, always affected the results in the expected direction, *i.e. *larger litters produced smaller and less agile pups. This again supports that the statistical methods employed would detect subtle changes where these were in fact present. Thus, any effects of PCB153 larger than the effect of litter size would presumably be detected.

### Animal model

Interpretations of the open field data at weaning were ambiguous, and the results did not provide a good indication of effects on anxiety from diet or dose PCB. The interpretation of open field data must be performed with care [75]. The ambiguous results could be influenced by the immature exploratory behaviour in pre-weanling mice [64], but may also reflect the nature of BALB/c mice used. BALB/c mice are phenotypically neophobic [76], which may have reduced the degree of exploration and range of behaviour displayed in the open field, thus limiting the possibilities to discern effects of exposure among groups. Selection of a model with higher reactivity to the test, such as the less anxious C57BL/6 might have improved the ability to discern effects among groups.

### Statistical methods

Above all, this study highlights the importance of taking litter size into consideration in statistical evaluations of neurodevelopmental responses. The findings support previous observations showing that the litter effect may be large and should always be included as a confounding factor or predictor of the responses [7,67,68]. Not taking into account the effects of intra-litter correlation and the litter size, may give an erroneous impression of group differences that are in reality caused by differences in litter size and maternal care [68]. Particularly in studies of teratogenic effects, or after maternal exposure of pups, the litter effect may be prominent. It is essential to keep in mind that the rearing condition (litter size) and the toxic exposure (dose) of each litter are mutually dependent and are defined by the maternal care provided by each individual dam. Including both litter size and dam in the analyses is therefore essential to discriminate between the individual effects, and will also provide a statistical measure on the magnitude of the litter effect.

If any effects of PCB153 were indeed present and of the same magnitude as the effect of litter size it would have been detected, even with sample sizes as low as three animals. The benefit of the factorial design is that it artificially increases the sample size when groups are combined for investigation of the factor they have in common (diet, exposure level or litter size). This means that even though sample size is low for some groups, the total number of litters exposed to *i.e. *"high level" is the sum of Fish High PCB and Casein High PCB together, which is 5+6 = 11 litters. Thus the statistical power for detecting effects of a high dose PCB153 is increased compared to using a one-way ANOVA where the groups are investigated separately. For the factor "litter size" it means investigating all litters with the same number of pups, regardless of diet or dose, to see if they are more similar than litters of different sizes. The low sample size however obstructs detection of differences among groups and the potentially ameliorating effects of maternal nutrition. Although reproductive output in this study was lower than expected, the differences in reproduction appeared not to be related to PCB153 exposure.

### Behavioural and cognitive effects of PCB153

It seems to be a recurring finding that organohalogen exposure at low doses that do not affect growth or physical development, still produce aberrant behaviour at later stages [11-13,17,59]. As concerns the present exposure level, the repeated doses to the dams were in the range of the single exposure doses (1.4 μmol/kgBW*day) that have been shown to produce aberrant behavioural effects after a single oral dose on PND10 [12,13,56]. Thus it was assumed that pups in the present study were exposed to sufficiently high levels during the vulnerable phase to potentially produce effects. Analyses of tissue levels in pups showed that they had high levels of PCB153 in liver and fat on PND19, indicating a high exposure to this di-ortho PCB congener throughout the period of BGS. The concentrations of PCB in the brains of pups which showed aberrant behaviour in previous studies [17] were low at the time of behavioural testing. The current results suggest, however, that the effects are greater with exposure to a single, high bolus dose around BGS than with continuous exposure or slow accumulation throughout gestation and lactation.

Neurotoxicity of the di-ortho PCBs have been shown to manifest with age [10,12,13], but in the present study, no significant effects in cognitive function or sensorimotor gating were seen. However, the trends to difference in PPI, the observed increase in male aggression throughout the trial and a corresponding lack of habituation in the ASR test of the PCB exposed groups, indicate an increased stress level or increased anxiety in males. The lack of habituation in the ASR-test may also be interpreted as a lack of ability to comprehend and adapt to the novel situation which the startle-box represents. The study of ASR/PPI needs to be repeated with a larger sample size for conclusions regarding the observations of lower PPI in PCB153 exposed males.

### Biochemical effects of PCB153

In previous epidemiologic studies of thyroid hormone disruption, exposure to the background levels of persistent organic pollutants (POPs) in Scandinavia had little effect on thyroid hormones in breast-fed infants [77]. In contrast, severe thyroid hormone disruption and impacts on cognitive function were seen after consumption of highly PCB contaminated rice-oil [73], potentially containing traces of furanes and unidentified contaminants as well as PCB. A combination of mixture-effects and exposure levels is likely to be of importance to the thyroid disrupting effects seen in other studies, whereas the PCB153 congener alone at the present doses did not affect the thyroid hormone levels or the blood parameters investigated. Only a slight difference in TG levels was seen among females. However, the TG levels were seemingly reduced by PCB exposure, which is in contrast to previous findings [78] where exposure to a mixture of POPs increased the levels of TG in rats. Since the TG levels in males showed no consistent differences related to PCB153 exposure, the higher TG and also HSI in Fish Control females may be explained by the higher body weight

### Human relevance of the study regarding neonatal PCB exposure

The findings imply that resource availability, nutrition, maternal care and social environment during gestation and early development affect development and behaviour to a much larger extent than the present exposure to PCB153. In mice these parameters are defined by the litter size. This suggests that the developmental starting point may outweigh other influences. The importance of rearing conditions and nutrient on development and behaviour has recently been reviewed [58]. The importance of maternal care and nutrition also supports recent studies in children [37], where Docosahexaenoic Acid (DHA) and selenium, as well as breastfeeding were found to protect against adverse effects of environmental exposure in early childhood [37]. Breastfeeding and maternal care have also been suggested to modify the effects of contaminants in human behavioural development [79].

## Conclusions

Exposure of mice offspring to the single congener PCB153 via the dam did not result in effects on physical development and behaviour that exceeded the biological influence of litter size. Synchronization of development such as eye opening and incisor eruption may have been compromised by neonatal PCB exposure. A maternal fish diet increased pup body weight and accelerated physical development, but did not alter pup reflex development or behaviour to a great extent. Although a larger sample size would increase the statistical power and would help detect differences, it was shown that random variables, such as litter size have a great impact on pup development and behaviour. This needs to be considered in experimental design and statistical analyses. The study demonstrated that subtle differences can be detected using the current methods and statistical treatment of the data.

## Competing interests

The authors declare that they have no competing interests.

## Authors' contributions

MHA took active part in designing the project, preparing the experimental diets, animal housekeeping, collecting behavioural data, tissue sampling; performing the statistical analyses, writing the original draft and completing the manuscript. ABE took active part in preparation of the experimental diets, animal housekeeping, data collection and statistical analyses and performing the behavioural tests. FJE was involved in selection and design of the ASR-PPI and the analysis of ASR-PPI-data. EHE selected, implemented and validated the statistical models using the R-software. TBR and AKL took part in designing the study and in revising and proofreading the manuscript. All authors read and approved the final manuscript.

## Supplementary Material

Additional file 1 **Composition of experimental diets**. Table of dietary components in the fish and casein based diets, given as g/kg feed. Click here for file

Additional file 2 **Analysed fat and protein content for individual diets**. Table showing the mean protein and fat content of two parallel samples analysed by accredited methods at NIFES. Click here for file

Additional file 3 **Test regime for the Acoustic Startle Response (ASR) with Prepulse Inhibition**. Table showing the chronological setup of the ASR/PPI experiment, with the sound pressure levels and pseudorandomized order of prepulse stimuli. Click here for file

Additional file 4 **Prepulse inhibition in male mice week 16**. Figure showing the inhibition of the acoustic startle response with increasing prepulse intensities in male mice on week 16. Click here for file

Additional file 5 **Percent survival in male pups from week 4 to 16**. Figure showing the survival and mortality of male mice from week 4-16 after exposure to PCB153 during gestation and lactation. Click here for file

Additional file 6 **Liver enzymes, glucose and blood lipids in serum from females and males in week 16**. Table of analysed concentrations of liver enzymes, glucose and blood lipids in serum from male and female mice in week 16, after exposure to PCB153 during gestation and lactation. Click here for file

Additional file 7 **Concentrations of nutrients in the experimental feeds**. Table of analysed concentrations of protein, fat and selected vitamins and fatty acids in the casein- and fish-based diets. Click here for file
