# Supplementary material for: Long-term effects of environmentally relevant doses of 2,2',4,4',5,5' hexachlorobiphenyl (PCB153) on neurobehavioural development, health and spontaneous behaviour in maternally exposed mice
Source: Behav Brain Funct. 2011 Jan 13;7:3. doi: 10.1186/1744-9081-7-3 (PMC3033814; doi:10.1186/1744-9081-7-3)
Supplement: Additional file 1 — Composition of experimental diets. Table of dietary components in the fish and casein based diets, given as g/kg feed. [file 1744-9081-7-3-S1.DOCX]

**Additional file 1 - Composition of experimental diets.**

|  |  | |
| --- | --- | --- |
| **Feed ingredient** | **Casein-diets**  **g/kg feed** | **Fish-diets g/kg feed** |
| Freeze dried salmon fillet* | 0 | 150 |
| Casein (89% protein) | 191 | 92.6 |
| Vitamin and mineral mix | 200.5 | 200.5 |
| Soy-bean oil | 100 | 44.7 |
| Dextrin | 508.5 | 512.3 |
| TOTAL (g/kg feed) | 1000 | 1000 |

Diets were made to meet 1995 NRC rat/mouse reproduction, gestation and lactation values (DYETs Inc. formulation #110800). Fish based diets were supplemented with casein and soy-bean oil to reach the same levels of protein and fat as the casein based diets. All ingredients were weighed before addition to the feeds. The amount of dextrin added was adjusted to maintain the correct percentage protein and fat in each diet.

*The Atlantic salmon was experimentally raised on plant protein and vegetable oils.
