# Supplementary material for: Long-term effects of environmentally relevant doses of 2,2',4,4',5,5' hexachlorobiphenyl (PCB153) on neurobehavioural development, health and spontaneous behaviour in maternally exposed mice
Source: Behav Brain Funct. 2011 Jan 13;7:3. doi: 10.1186/1744-9081-7-3 (PMC3033814; doi:10.1186/1744-9081-7-3)
Supplement: Additional file 2 — Analysed fat and protein content for individual diets. Table showing the mean protein and fat content of two parallel samples analysed by accredited methods at NIFES. [file 1744-9081-7-3-S2.DOCX]

**Additional file 2- Analysed fat and protein content for individual diets**

| Diet | Protein (%) | Fat (%) |
| --- | --- | --- |
| Casein Control | 17.8 | 10.1 |
| Fish Control | 17.9 | 10.4 |
| Casein Low PCB | 17.9 | 10.1 |
| Fish Low PCB | 17.6 | 10.3 |
| Casein High PCB | 17.4 | 9.8 |
| Fish High PCB | 17.4 | 10.5 |

Reported values represent mean of 2 parallels analysed by accredited methods at NIFES.
