# Supplementary material for: Long-term effects of environmentally relevant doses of 2,2',4,4',5,5' hexachlorobiphenyl (PCB153) on neurobehavioural development, health and spontaneous behaviour in maternally exposed mice
Source: Behav Brain Funct. 2011 Jan 13;7:3. doi: 10.1186/1744-9081-7-3 (PMC3033814; doi:10.1186/1744-9081-7-3)
Supplement: Additional file 3 — Test regime for the Acoustic Startle Response (ASR) with Prepulse Inhibition. Table showing the chronological setup of the ASR/PPI experiment, with the sound pressure levels and pseudorandomized order of prepulse stimuli. [file 1744-9081-7-3-S3.DOCX]

**Additional file 3 – Test regime for the Acoustic Startle Response with Prepulse Inhibition**

| Block | Trial | Prepulse (20 ms) | Stimulus (40 ms) | *For calculation of :* |
| --- | --- | --- | --- | --- |
| **1** | 1-5 | no prepulse | 120 dB | *Habituation* |
| **2** | 1 | 75dB | 120 dB | *ASR-pp* |
|  | 2 | no prepulse | 120 dB | *ASR-simple* |
|  | 3 | 85dB | 120 dB | *ASR-pp* |
|  | 4 | 73dB | 120 dB | *ASR-pp* |
|  | 5 | no prepulse | 120 dB | *ASR-simple* |
|  | 6 | 80dB | 120 dB | *ASR-pp* |
|  | 7 | no prepulse | 120 dB | *ASR-simple* |
|  | 8 | 80dB | 120 dB | *ASR-pp* |
|  | 9 | 73dB | 120 dB | *ASR-pp* |
|  | 10 | no prepulse | 120 dB | *ASR-simple* |
|  | 11 | 85dB | 120 dB | *ASR-pp* |
|  | 12 | 75dB | 120 dB | *ASR-pp* |
|  | 13 | 73dB | 120 dB | *ASR-pp* |
|  | 14 | no prepulse | 120 dB | *ASR-simple* |
|  | 15 | 80dB | 120 dB | *ASR-pp* |
|  | 16 | 85dB | 120 dB | *ASR-pp* |
|  | 17 | no prepulse | 120 dB | *ASR-simple* |
|  | 18 | 75dB | 120 dB | *ASR-pp* |
|  | 19 | 73dB | 120 dB | *ASR-pp* |
|  | 20 | 75dB | 120 dB | *ASR-pp* |
|  | 21 | no prepulse | 120 dB | *ASR-simple* |
|  | 22 | 85dB | 120 dB | *ASR-pp* |
|  | 23 | no prepulse | 120 dB | *ASR-simple* |
|  | 24 | 80dB | 120 dB | *ASR-pp* |
|  | 25 | 85dB | 120 dB | *ASR-pp* |
|  | 26 | no prepulse | 120 dB | *ASR-simple* |
|  | 27 | 80dB | 120 dB | *ASR-pp* |
|  | 28 | no prepulse | 120 dB | *ASR-simple* |
|  | 29 | 73dB | 120 dB | *ASR-pp* |
|  | 30 | 75dB | 120 dB | *ASR-pp* |
| **3** | 1-5 | no prepulse | 120 dB | *Habituation* |

ASR = Acoustic Startle response; ASR-simple: ASR without prepulse, ASR-pp: ASR with a prepulse 73-85dB; dB= decibel. The prepulses were presented 100ms prior to the main (120dB) stimulus, and the peak startle response was recorded. White noise was used for all stimuli.
