# Supplementary material for: Long-term effects of environmentally relevant doses of 2,2',4,4',5,5' hexachlorobiphenyl (PCB153) on neurobehavioural development, health and spontaneous behaviour in maternally exposed mice
Source: Behav Brain Funct. 2011 Jan 13;7:3. doi: 10.1186/1744-9081-7-3 (PMC3033814; doi:10.1186/1744-9081-7-3)
Supplement: Additional file 4 — Prepulse inhibition in male mice week 16. Figure showing the inhibition of the acoustic startle response with increasing prepulse intensities in male mice on week 16. [file 1744-9081-7-3-S4.DOCX]

**Additional file 4 – Prepulse inhibition in male mice in week 16.**

Inhibition of the startle response with increasing prepulse intensities of 73dB (upper left), 75dB (upper right), 80dB (lower left) and 85dB (lower right). The bars and the error bars represent the percent prepulse inhibiton (mean +/**-** SEM). A high negative value (*i.e.* -60%) indicates that the animals showed the normal response with reduced startle amplitude after presentation of a prepulse stimulus, whereas a low negative value (*i.e. -10%*) means little difference in startle response with presentation of a prepulse. Sample sizes were (females/males): Fish Control: n=3/3; Fish High: n=4/5; Casein high, n=6/4.
