# Supplementary material for: Long-term effects of environmentally relevant doses of 2,2',4,4',5,5' hexachlorobiphenyl (PCB153) on neurobehavioural development, health and spontaneous behaviour in maternally exposed mice
Source: Behav Brain Funct. 2011 Jan 13;7:3. doi: 10.1186/1744-9081-7-3 (PMC3033814; doi:10.1186/1744-9081-7-3)
Supplement: Additional file 5 — Percent survival in male pups from week 4 to 16. Figure showing the survival and mortality of male mice from week 4-16 after exposure to PCB153 during gestation and lactation. [file 1744-9081-7-3-S5.DOCX]

**Additional file 5 – Percent survival in male pups from week 4 to 16.**


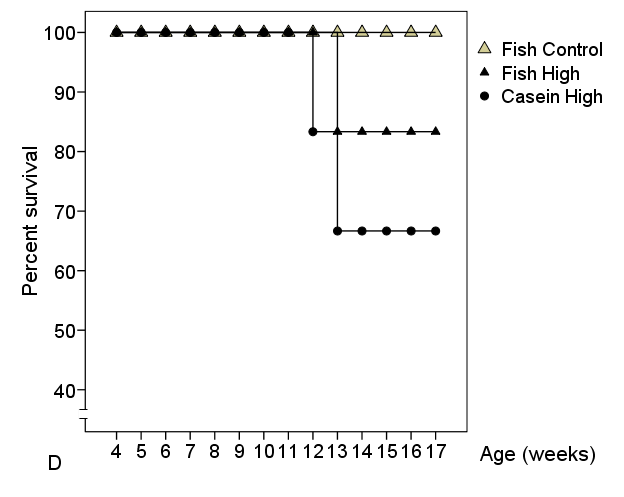


Survival of male BALB/c mice exposed to PCB153 via gestation and lactation. Fish Control: n=3; Fish High: n=5; Casein High n=6.
