# Supplementary material for: Long-term effects of environmentally relevant doses of 2,2',4,4',5,5' hexachlorobiphenyl (PCB153) on neurobehavioural development, health and spontaneous behaviour in maternally exposed mice
Source: Behav Brain Funct. 2011 Jan 13;7:3. doi: 10.1186/1744-9081-7-3 (PMC3033814; doi:10.1186/1744-9081-7-3)
Supplement: Additional file 6 — Liver enzymes, glucose and blood lipids in serum from females and males in week 16. Table of analysed concentrations of liver enzymes, glucose and blood lipids in serum from male and female mice in week 16, after exposure to PCB153 during gestation and lactation. [file 1744-9081-7-3-S6.DOCX]

**Additional file 6 – Liver enzymes, glucose and blood lipids in serum from females and males in week 16**

| Gender | Group | N | ALP (Units/L) | LDH (Units/L) | Glc (mmol/L) | HDL (mmol/L) | Chol (mmol/L) | TG (mmol/L) |
| --- | --- | --- | --- | --- | --- | --- | --- | --- |
| Females | Fish Control | 3 | 249.00±20.50 | 397.67±44.80 | 14.35±0.72 | 3.12±0.25 | 3.54±0.22 | 2.10±0.05^a^ |
|  | Casein High PCB | 5 | 262.60±11.53 | 500.60±30.36 | 14.32±0.74 | 2.73±0.14 | 2.99±0.20 | 1.51±0.14^b^ |
|  | Fish High PCB | 4 | 285.50±9.02 | 423.50±27.27 | 14.02±1.00 | 2.57±0.10 | 2.88±0.12 | 1.52±0.09^ab^ |
| Males | Fish Control | 3 | 273.00±21.00 | 376.00±50.85 | 14.85±0.39 | 3.20±0.27 | 6.18±2.04 | 2.13±0.38 |
|  | Casein High PCB | 4 | 240.75±12.99 | 602.25±73.69 | 13.60±0.62 | 3.48±0.12 | 4.16±0.31 | 2.41±0.51 |
|  | Fish High PCB | 4 | 248.00±17.83 | 450.25±36.26 | 13.12±1.03 | 3.40±0.12 | 4.31±0.24 | 1.48±0.39 |

The animals were exposed to PCB153 throughout gestation and lactation, via maternal consumption of spiked feeds based on fish or casein. High dose feeds (Fish High PCB and Casein High PCB) contained ~1500µg PCB153/kg feed. Data are presented as mean±SEM. ALP=Alkaline Phosphatase, LDH=Lactate dehydrogenase, Glc=Glucose, HDL=High Density Lipoprotein cholesterol, Chol=Total Cholesterol, TG=triglycerides. Groups with different superscript letters are significantly different within the same gender (Mann-Whitney U, p<0.05).
