# Supplementary material for: Long-term effects of environmentally relevant doses of 2,2',4,4',5,5' hexachlorobiphenyl (PCB153) on neurobehavioural development, health and spontaneous behaviour in maternally exposed mice
Source: Behav Brain Funct. 2011 Jan 13;7:3. doi: 10.1186/1744-9081-7-3 (PMC3033814; doi:10.1186/1744-9081-7-3)
Supplement: Additional file 7 — Concentrations of nutrients in the experimental feeds. Table of analysed concentrations of protein, fat and selected vitamins and fatty acids in the casein- and fish-based diets. [file 1744-9081-7-3-S7.DOCX]

**Additional file 7 - Concentrations of nutrients in experimental feeds**

| **Nutrient** | **Casein Control**  **n=2** | **Fish Control**  **n=2** |
| --- | --- | --- |
| Protein (%) | 17.7±0.6 ^a^ | 17.6±0.3^a^ |
| Fat (%) | 10.0±0.2^a^ | 10.4±0.2^a^ |
| Monounsaturated FA (mg/g) | 24.0 | 37.6 |
| Polyunsaturated FA (mg/g) | 60.3 | 44.9 |
| LA (18:2n-6) (mg/g) | 53.3 | 31.3 |
| ALA (18:3n-3) (mg/g) | 6.9 | 6.8 |
| EPA (20:5n-3) (mg/g) | 0.0 | 1.20 |
| DHA (22:6n-3) (mg/g) | 0.0 | 2.4 |
| SUM n-3 (mg/g) | 7.1 | 12.5 |
| SUM n-6 (mg/g) | 53.3 | 32.4 |
| Ratio (n-3/n-6) (mg/g) | 0.1 | 0.4 |
| Vitamin A (mg/kg) | 0.5 | 1.1 |
| Vitamin D3 (mg/kg) | 0.03 | 0.03 |
| Vitamin E (mg/kg) | 178.0 | 172.0 |

Table showing the mean of two analysed parallel samples per control diet. All diets were made with identical vitamin supplements. FA=Fatty Acid; LA=Linolenic Acid; ALA=Alpha Linolenic Acid; EPA=Eicosapentaenoic Acid; DHA=Docosahexaenoic Acid; n-3=Omega-3 FA; n-6=omega-6 FA; Vitamin A=Sum Retinol; Vitamin E=alpha-tocopherol.

^a^: All the experimental diets (n=6) were analysed for content of protein and fat, and the mean±SD includes analyses of all the experimental diets.
